# Help-Seeking Behavior of Adults with Adverse Childhood Experiences in Rural China

**DOI:** 10.3390/bs16050818

**Published:** 2026-05-19

**Authors:** Weizhi Chen, Yiran Zhang, Jinyu Chen

**Affiliations:** 1 Department of Social Work, Qingdao University of Science and Technology, Qingdao 266061, China; 2 School of Political Science and Public Administration, Shandong University, Qingdao 266000, China; 3 School of Humanities and Social Science, Yanbian University, Yanji 133002, China

**Keywords:** help-seeking behavior, adverse childhood experiences, rural China, theory of planned behavior

## Abstract

Adverse Childhood Experiences (ACEs), which encompass a broad range of adverse events during childhood, are prevalent in rural China. However, help-seeking among adults with ACEs remains limited and underexplored. This study aims to examine the barriers to help-seeking behaviors among adults with ACEs in rural China. Qualitative interviews were conducted with 20 adults affected by ACEs in rural mainland China between October 2024 and December 2024. Data were collected through semi-structured interviews in Mandarin, and transcripts were analyzed using the Theory of Planned Behavior (TPB) framework, focusing on behavioral beliefs, normative beliefs, and perceived behavioral control. The findings reveal barriers to help-seeking among individuals with ACEs in rural China, categorized into three key dimensions: (1) Behavioral Beliefs: Beliefs that corporal punishment is the responsibility as well as love of parents, and misconceptions attributing ACEs to personal faults significantly hindered help-seeking. (2) Normative Beliefs: Respect for parental authority in China culture context hinder help-seeking for adults with ACEs. Moreover, gender differences were evident, with men avoiding help-seeking due to perceived shame, while women were more likely to confide in friends and family. Finally, stigmatization of mental health services further inhibited help-seeking behaviors. (3) Perceived Behavioral Control: The lack of formal and informal support systems in rural areas exacerbated the issue, highlighting significant gaps in resource accessibility and cultural acceptance of mental health support. Addressing these barriers through public education, destigmatization of mental health services, and improved resource allocation could facilitate help-seeking behaviors and improve outcomes for individuals affected by ACEs.

## 1. Introduction

Adverse Childhood Experiences (ACEs) refer to negative childhood events of varying severity that occur within a child’s family or social environment (Kalmakis & Chandler, 2014). These include experiences such as child abuse, household dysfunction, and neglect (Felitti et al., 1998; Anda et al., 1999). Research indicates that between 50% and 64% of adults in the United States report having experienced ACEs (Merrick et al., 2018). Extensive studies have highlighted the detrimental effects of ACEs on adults, particularly in physical, mental, and behavioral health domains, in both Western and Chinese societies (Petruccelli et al., 2019; L. Zhang et al., 2020). For example, trauma-based perspectives suggest that individuals exposed to chronic childhood adversity may develop dissociative and cognitive adaptation mechanisms, which can affect emotional regulation, identity formation, and coping behaviors (Ozturk, 2025).

Social services have the potential to mitigate the negative physical and mental health outcomes associated with ACEs (Mueller-Coyne et al., 2024). However, in the Chinese context, individuals often refrain from seeking help (Mok et al., 2008). Mental health services in rural China are severely limited (L. Liu et al., 2018), and cultural norms discourage help-seeking, often associating it with shame and stigma (Rudowicz & Au, 2001). There remains a significant gap in understanding with regard to the factors that influence help-seeking behaviors in response to ACEs in rural China. This study aims to address this gap by exploring the unique barriers to help-seeking in rural China.

### 1.1. Adverse Childhood Experiences

ACEs encompass a broad range of negative childhood experiences, including abuse, neglect, exposure to domestic violence, living in a stressful household, or cohabiting with family members who misuse alcohol or drugs (World Health Organization, 2016). This study focuses on ACEs related to child maltreatment occurring within family or school settings. Both families and schools play a significant role in influencing health outcomes associated with ACEs (Balistreri & Alvira-Hammond, 2016; Forster et al., 2020), and numerous studies have highlighted the negative outcomes of ACEs on adults’ physical and mental health, particularly in Western societies (Petruccelli et al., 2019; Kalmakis & Chandler, 2015). For instance, a review of 37 studies found that ACEs significantly increase the risk of negative health outcomes (Hughes et al., 2017). Similarly, a scoping review of 58 studies revealed that ACEs adversely affect mental health and social functioning (Tzouvara et al., 2023).

ACEs are also prevalent in China (L. Zhang et al., 2020), particularly in rural areas. One study found that three-fourths of Chinese youth reported experiencing at least one of the ten conventional ACEs (L. Zhang et al., 2020). Moreover, ACEs have been linked to poor health outcomes in China. For example, studies have shown associations between ACEs and chronic diseases (Lin et al., 2021) as well as suicidal behaviors (S. Li et al., 2021). Some ACEs, such as corporal punishment and humiliation, are culturally accepted in China (Tang, 2006; Fung, 1999), which may exacerbate the negative outcomes associated with ACEs (Ho et al., 2019).

### 1.2. Help-Seeking with ACEs

Help-seeking can occur at various stages of life (Cornally & McCarthy, 2011). This study focuses on the actions taken by adults to seek assistance for ACEs. In Western societies, help-seeking is relatively common and proactive (Malashevsky, 2024), with individuals seeking both professional and informal support (Karatekin, 2024). For instance, studies have emphasized the critical role of mental health services, such as University Psychological Counseling, in supporting students who have experienced ACEs (Craig et al., 2023; Almogheer, 2024). Additionally, informal strategies, such as masking emotions, self-reliance, and sharing experiences, are widely used (Lester et al., 2019).

Previous studies in Western cultures have indicated that help-seeking behaviors are influenced by individual characteristics (Galdas et al., 2005). For example, a systematic review of 40 studies found that socio-demographic factors significantly affect help-seeking behavior (Magaard et al., 2017). Perceived need is another critical factor (Gonzalez et al., 2005) that influences help-seeking. For instance, a study based on multiple regression analyses found that perceived benefits and barriers directly predict help-seeking behavior (O’Connor et al., 2014). Most previous studies have used quantitative approaches to explore risk factors without considering participants’ perceptions and experiences. Additionally, the differences between Chinese and Western cultural contexts may contribute to variations in the experience of ACEs (Ho et al., 2019) and help-seeking behaviors (Z. Liu et al., 2014). For example, corporal punishment and emotional abuse are more culturally accepted in China (Hou et al., 2011; Cui & Liu, 2020), which could impact help-seeking behaviors. Furthermore, rural communities in China often lack access to mental health services (C. Deng et al., 2023; L. Liu et al., 2018), which can pose significant barriers to seeking help for ACEs.

### 1.3. Theory of Planned Behavior Framework

The Theory of Planned Behavior (TPB), proposed by Ajzen (1991), suggests that behavior is influenced by three components: attitude, subjective norm, and perceived behavioral control. Attitude refers to an individual’s evaluation of the positive or negative outcomes of a behavior, while subjective norm represents the perceived social pressure to engage in the behavior. Perceived behavioral control pertains to an individual’s perception of their ability to perform the behavior, influenced by both internal and external factors. These components collectively shape behavioral intention, which directly impacts actual behavior. A positive attitude and strong subjective norm can increase the intention to engage in a behavior, while higher perceived behavioral control can enhance confidence and readiness. Thus, intention results from the interaction of these factors, with perceived behavioral control potentially influencing both intention and behavior directly, particularly when individuals feel they lack control over the behavior (Ajzen, 1991). TPB has been widely used to understand child-related behaviors (Walsh et al., 2009). For example, a meta-analysis based on the TPB framework found that attitudes, subjective norms, and perceived behavioral control predict parental involvement in child health behavior (Hamilton et al., 2020).

### 1.4. The Current Study

While help-seeking behaviors in Western societies are well-documented and supported by abundant resources such as psychological counseling (Karatekin, 2024), the factors influencing help-seeking behaviors for ACEs in rural China remain ambiguous. In rural China, the lack of mental health resources (C. Deng et al., 2023) and the cultural acceptance of child abuse complicate the process of seeking help (Wong, 2019; Mok et al., 2008). However, to our knowledge, no previous research gave the adults to voice to express their experience in help-seeking or provided an understanding of the help-seeking of ACEs from the perspective of adults in rural China. This study aims employ a qualitative method to explore help-seeking barriers among adults with ACEs, providing insights into the unique challenges faced in this context.

## 2. Method

This study employs a qualitative research design and utilizes individual semi-structured interviews to explore the experiences of adults who have encountered ACEs in rural China.

### 2.1. Sampling and Recruitment

The study recruited 20 participants who had experienced ACEs at a local community center in rural China. A purposive sampling method was used to identify adults with ACEs through community-based verbal referrals. This was followed by snowball sampling, in which participants were invited to verbally refer other individuals who met the study criteria. All participants were required to meet the following inclusion criteria: (1) individuals who experienced ACEs related to child maltreatment occurring within family or school settings; (2) individuals who experienced ACEs before the age of 18 and are now over the age of 18; (3) individuals currently residing in rural mainland China.

The research team initially contacted potential participants via WeChat or telephone to explain the study’s purpose and provide relevant information. Potential participants were then instructed to contact the research team if they were willing to participate. After confirming their willingness, the research team scheduled the interview and confirmed the date and time. Each interview lasted approximately 30 min, with one participant’s interview extending to 60 min. The demographic characteristics of the participants are presented in Table 1.

### 2.2. Ethics

Ethical approval was obtained from the Technology Ethics Committee at Qingdao University of Science and Technology (QKDDL-2024-50). Prior to the interview, each participant received and signed a digital consent form outlining the study’s aims, procedures, potential risks, and benefits.

### 2.3. Data Collection

Between October 2024 and December 2024, semi-structured interviews were conducted with 20 adults who experienced ACEs. The third author conducted all interviews with the participants. Before the interviews, the researcher explained the definition of ACEs and the study’s inclusion criteria to the participants to ensure their understanding of the relevant concepts, and eligibility for participation was confirmed based on participants’ self-reports. Each interview lasted between 30 and 60 min and was conducted in Mandarin Chinese. All interviews were audio-recorded with the participants’ permission and were guided by a semi-structured interview protocol. Interviews were conducted either face-to-face (*n* = 14) or via WeChat (*n* = 6).

Researchers underwent training on the ethical aspects of human subjects research prior to data collection. Interviews were recorded using a digital voice recorder, with the informed consent process ensuring that participants were fully aware of the guidelines and any potential risks or benefits associated with participation.

### 2.4. Data Analysis

Ajzen’s (1991) TPB was used as a guiding theoretical framework for developing the semi-structured interview guide and interpreting participants’ help-seeking behaviors. This framework includes three key components: attitude, subjective norm, and perceived behavioral control, which collectively influence intentions and, ultimately, behaviors. The adapted model provided a structured approach to understanding participants’ help-seeking behaviors in a culturally relevant manner.

Following each interview, the researchers transcribed all interviews verbatim in Chinese. To ensure accuracy, participants were provided with the transcriptions for review, though none requested changes. The transcriptions were then analyzed using thematic analysis. In the open coding phase, relevant information was identified and assigned preliminary codes. In the axial coding phase, similar initial codes were grouped to form higher-level categories. Finally, during the selective coding phase, core themes influencing help-seeking behaviors were identified.

The coding process was conducted in Chinese to preserve the cultural and linguistic nuances of the participants’ responses. Weekly meetings were held to review feedback, share insights, and address any discrepancies between the coders. To ensure consistency, a final review of all codes was conducted by a third researcher.

## 3. Results

In rural China, many participants with ACEs did not report help-seeking behaviors. They believed that ACEs from family were culturally acceptable and an expression of parental responsibility. Additionally, Chinese traditional culture hindered help-seeking, as many participants lacked access to support resources and social networks, which prevented them from seeking help. Through interviews, this study identifies three key dimensions of help-seeking behavior: behavioral beliefs, normative beliefs, and perceived behavioral control.

### 3.1. Behavioral Beliefs

#### 3.1.1. Belief About Parenting Behavior: Love and Responsibility

Many participants believed that “corporal punishment is a form of education and also a manifestation of parental love.” For example, Du, who experienced harsh discipline from her mother in childhood, recalled telling her mother, “I wish you would die.” However, as an adult, Du reflected that her mother’s corporal punishment was necessary:

*“I understand now that my parents’ discipline was actually out of love. My mother was so busy and tired, yet she still found time to correct my flaws.”*

All participants viewed corporal punishment as a sign of responsible parenting. One participant stated: “To ensure that children succeed in the future, harsh discipline is necessary in childhood. Not punishment a child is neglectful parenting.” For example, Yong believed that his father’s physical punishment was a way to educate him because he had been particularly mischievous:

*“I thought my dad’s beating was a form of education. I used to be very naughty, if my dad hadn’t hit me, I wouldn’t have learned properly. He was responsible for educating me.”*

#### 3.1.2. Belief About Self-Behavior: Self-Blame

Many participants exhibited self-blame, attributing the corporal punishment they experienced in childhood to their own behavior or academic difficulties. They believed that ACEs were their fault, even when they endangered their safety. For instance, Yong thought he was punished because he made unreasonable demands on his parents:

*“I took advantage of being sick to demand things I couldn’t normally get. For example, I told them if they didn’t buy me something, I wouldn’t go to school, so he had no choice but to hit me.”*

After attributing the cause of ACEs to themselves, many participants further indicated that they lacked the ability to manage their emotions, believing that the trauma from ACEs should be dealt with privately rather than through help-seeking. Dan, who was abused by her father, stated:

*“I probably wouldn’t think my father’s beating was his problem, but rather, my problem. It’s my inability to live happily.”*

### 3.2. Normative Beliefs

#### 3.2.1. Respect for Parental Authority

Many participants refused to involve authorities, such as the police, when experiencing physical abuse. As one participant noted, “After all, he is my father.” Seeking external help was perceived as a sign of disrespect toward parents, which prevented adults from seeking help even in the face of ACEs. Tao, who, along with his mother, suffered long-term domestic violence from his father, explained:

*“I never thought about calling the police because after all, he is my father! If I did, the family would fall apart.”*

Many participants reported that parents held significant authority in the household, and family members were expected to comply. For example, Dan chose to endure corporal punishment silently, saying:

*“He’s an elder, and there’s this inherent respect for him. No matter what he does, it’s hard to develop feelings of resistance.”*

#### 3.2.2. Masculinization

Masculinity also influenced the participants’ reluctance to express their ACEs. Many male participants believed that the male image in Chinese culture is one of being “stoic” and “not shedding tears easily,” which made it harder for them to express vulnerability. Tao explained:

*“I feel it would be embarrassing to talk about it (ACEs). It feels like everyone would laugh at you, even though they wouldn’t openly ridicule you.”*

Only one male participant actively spoke about his ACEs, while the majority remained silent. In contrast, some female participants reported feeling more comfortable sharing their ACEs with female friends, which provided them with emotional support. For example, Yue said: “I talk about my ACEs to my friends, and it really makes me feel much better.”

#### 3.2.3. Stigma

Many participants equated mental health problems with “illness,” which deterred them from seeking help, even when they were suffering from depression or suicidal thoughts due to ACEs. Dong believed that counseling was only for those with severe mental illnesses:

*“I always thought only people who are ill go there (receive mental health services).”*

Mental health services in rural China are often stigmatized, with many believing that individuals who seek these services are “mentally ill.” Ming, for example, thought he did not need mental health services:

*“People who go to those centers must be mentally unhealthy or have mental illnesses. I think I’m fine.”*

Participants also viewed seeking mental health services as an indicator of suicidal tendencies. Even those who did access mental health services were reluctant to disclose it to their community. Guo shared her experience:

*“Everyone thought I went to see a counselor because I wanted to commit suicide. So, if you go to see a counselor, the whole village will gossip. If I were to go, I’d definitely go secretly.”*

### 3.3. Perceived Behavioral Control

#### 3.3.1. Barriers to Accessing Resources

All participants reported that they had never heard of mental health counseling services until the advent of the Internet. For instance, Tao only became aware of such services as an adult:

*“Growing up, I only saw such services depicted in television dramas. In my impression, mental health counseling was always portrayed as something only wealthy people could afford. Before that, I had never even heard of it.”*

Furthermore, mental health service providers in rural China are often inadequately trained. Some participants reported that their schools lacked the capacity to provide mental health support, and teachers rarely paid attention to students’ psychological needs. Dan explained:

*“Teachers rarely focused on individual students outside of their lessons. They only care about teaching. They were not aware of the concept of psychological services either. They would simply say, ‘Don’t hit your children,’ but in reality, they showed little concern about whether parents actually refrained from doing so.”*

Additionally, some participants indicated that ACE interventions were primarily focused on pharmacological treatments, which did not address the root causes of ACEs. Qin, who experienced foster care, developed anxiety as a result. However, her treatment only involved medication, with no inquiries into her ACEs:

*“In modern hospitals, it feels like an assembly line. I underwent a CT scan, blood tests, and several other examinations, as well as completing a questionnaire. The doctor only gave me a prescription and asked nothing else. The entire consultation lasted less than a minute.”*

#### 3.3.2. Lack of Informal Support

Participants reported that they lacked the opportunity to share their experiences, hindering help-seeking. Ping described how his busy work environment deprived him of the chance to seek help:

*“Most of the people I work with have low educational backgrounds, none of them know how to offer help. The work is from morning until night, with no time for rest, so no one has the space to talk deeply.”*

Other participants expressed a willingness to share their ACEs, but due to a lack of opportunities, they had never initiated such discussions. Han reported:

*“We are always focused on studying, doing homework, or playing, so there is no time or space to talk about these things.”*

Participants were often unwilling to seek help from friends, neighbors, or relatives, fearing it would burden them emotionally. Cui, for example, thought that confiding in friends would place an emotional burden on them:

*“If you tell others, they will think, ‘How can I help you?’ It adds an emotional burden on them.”*

Some participants also felt that friends, although empathetic, were unable to offer useful advice. Dong, who had been severely bullied, shared:

*“Others may provide emotional support, but what’s the point? No one can offer emotional support every day, nor can they give any practical advice.”*

## 4. Discussion

This study investigates the barriers influencing help-seeking behavior among adults with ACEs in rural China, employing the TPB framework. The findings identify misconceptions, cultural norms, stigma, and a lack of resources as barriers to seeking help. These findings contribute to the existing literature by addressing the unique challenges faced by adults in rural China when coping with ACEs. First, regarding behavioral beliefs, this study builds upon previous research by revealing that many adults misattribute ACEs to their own behavioral shortcomings, which impacts their willingness to seek help. Second, in normative beliefs, the study uncovers a gender discrepancy, with men being more likely to perceive help-seeking as a source of shame. Moreover, stigma as a normative belief further hinders help-seeking behaviors in rural China. Third, concerning perceived behavioral control, the study highlights that the lack of adequate resources for help-seeking reinforces limited help-seeking behaviors in rural Chinese communities. Finally, this study extends the applicability of the TPB framework within the cultural context of rural China.

In the domain of behavioral beliefs, our findings underscore the significant role of individual-level cognitive beliefs in shaping help-seeking behaviors. Previous studies have found that help-seeking is common in Western societies, where adults often seek both professional and informal assistance (Malashevsky, 2024; Karatekin, 2024). However, in Chinese society, our study identified prevalent cognitive misconceptions surrounding ACEs, which act as barriers to help-seeking. Many participants misattributed ACEs to personal failings, such as poor academic performance or disobedience, and perceived help-seeking as a sign of disrespect toward their parents. These findings align with traditional Chinese cultural values emphasizing parental authority, self-discipline, and personal responsibility (Xu, 2022), which may contribute to a tendency to endure trauma without seeking external support. In addition, from a trauma-based theoretical perspective, historical accounts have suggested that child maltreatment may have functioned to maintain emotional stability within families and to alleviate caregivers’ feelings of guilt (DeMause, 1998). Such behavioral beliefs may contribute to the normalization of ACEs by both parents and children, ultimately discouraging help-seeking behaviors.

Regarding normative beliefs, the findings reveal a gender discrepancy in help-seeking behaviors, which may further exacerbate barriers to seeking help. This result aligns with earlier research from the UK, which also found that men were less likely than women to seek professional help for mental health issues (Oliver et al., 2005; Y. Yu et al., 2015). Women were more willing to confide in friends and family, potentially due to gendered norms that associate femininity with emotional expressiveness and relational support (Kennedy & Smith, 2020). For men, as inheritors of the family lineage, societal expectations often place greater responsibility on them compared to women (Kennedy & Smith, 2020). As a result, men are more likely to perceive help-seeking as a source of shame. In summary, cultural norms that reinforce gender discrepancies significantly hinder the likelihood of seeking help.

The stigma surrounding mental health emerged as a pervasive barrier to help-seeking for ACEs. Previous studies have documented the prevalence of stigma in mental health treatment in China (Y. Deng et al., 2022; Ran et al., 2018), but there has been a lack of focus on help-seeking behaviors in rural areas. To our knowledge, this study is the first to specifically explore how mental health stigma hinders help-seeking for ACEs in rural China. Many adults with ACEs avoided mental health services due to fears of stigmatization within their rural communities. This stigma likely stems from the broader stigmatization of mental illness (S. Yu et al., 2018) and a lack of awareness about ACEs (C. Chen et al., 2022). Participants often conflated mental illness with ACEs, transferring the stigma associated with mental illness onto ACEs, which further discouraged help-seeking.

In the domain of perceived behavioral control, we found that access to mental health-related resources significantly impacts help-seeking behavior. In contrast to Western societies, where individuals can access both formal and informal support systems (Karatekin, 2024), communities in China often lack adequate access to services (L. Liu et al., 2018). This issue is particularly pronounced in rural areas, where mental health service providers are frequently nonexistent, and many participants were entirely unaware of available services (C. Deng et al., 2023). This disparity can be attributed to the urban–rural distribution of medical resources, which has been exacerbated by China’s urban–rural binary system. This system allows cities to exploit rural resources, thereby widening the gap between urban and rural areas (Y. Chen et al., 2014; T. Zhang et al., 2017). Moreover, although rural China, similarly to Western contexts, possesses a range of informal support resources (Lester et al., 2019), adults with ACEs in rural China made only limited use of these resources, as friends and neighbors were often able to provide only limited emotional support. This may be influenced by the family-centered culture in China, in which disclosing psychological distress to others is often perceived as shameful and potentially damaging to the family’s social image. As a result, individuals may selectively disclose only less severe aspects of their ACEs when seeking informal support (J. Chen et al., 2025; X. Li & Li, 2025). Together, these factors create significant barriers to help-seeking for adults with ACEs in rural China.

This study applies Ajzen’s (1991) TPB framework to examine help-seeking behaviors among adults in rural China. Previous studies have shown that attitudes and perceived behavioral control are significant predictors of help-seeking intentions (Hamilton et al., 2020; Adams et al., 2022). The theoretical contribution of this study lies in extending the TPB framework to the cultural context of rural China. Our findings suggest that behavioral beliefs, subjective norms, and perceived behavioral control are deeply shaped by culturally specific understandings of family authority, stigma, gender expectations, and rural structural inequalities within the Chinese context. Moreover, while the TPB framework assumes that individuals form help-seeking intentions through rational evaluations of behavioral outcomes (Ajzen, 1991), our findings further indicate that many participants normalized or rationalized ACEs and therefore did not perceive a need to seek help. This phenomenon may be associated with the influence of filial piety culture in China, in which ACEs are sometimes interpreted as expressions of parental care or responsibility (Ho et al., 2019), thereby challenging the rational assumption underlying the TPB framework. Therefore, future studies applying TPB to trauma-related help-seeking in rural settings should further contextualize the framework within broader cultural and structural conditions.

### 4.1. Limitations

While this study provides valuable insights into help-seeking behaviors among adults with ACEs in rural China, it has several limitations. First, the study did not offer a detailed classification of participants’ ACEs, failing to distinguish between different types of ACEs within families, such as physical and emotional abuse, as well as differences between individuals with high and low levels of ACE exposure. Since ACEs are multifaceted, future research should investigate how various types of childhood adversity uniquely influence help-seeking behavior. In addition, this study did not collect detailed information regarding overly indulgent or pampering parenting styles. Therefore, their potential influence on ACEs and help-seeking behaviors could not be examined. Future research should further explore how such culturally specific parenting practices may shape adversity experiences and subsequent help-seeking behaviors. Second, this study was limited to rural northern China, which may not fully represent rural areas across the country. Regional differences in attitudes toward ACEs and help-seeking behaviors likely exist, particularly between northern, southern, and western parts of China. Expanding future research to include other rural regions would allow for a more comprehensive understanding of these regional disparities and the challenges faced by adults with ACEs in rural China.

### 4.2. Implications

This study offers several important implications for policy and practice. At the national level, the findings highlight the importance of strengthening rural mental health systems by drawing on the World Health Organization’s Mental Health Gap Action Programme (WHO mhGAP; World Health Organization, 2008), which emphasizes expanding access to basic mental health services in low-resource settings. Such a framework can help address the shortage of mental health resources in rural China through system-level integration and capacity building. At the regional or service-delivery level, a trauma-informed care approach may be adopted to reduce stigma and promote gender-sensitive practice. By emphasizing safety, empowerment, and non-judgmental understanding of lived experiences, this approach can help challenge stigmatizing beliefs surrounding ACEs and encourage more inclusive and equitable mental health support. At the community level, task-shifting models can be used to enhance service accessibility by training community workers and non-specialists to provide basic psychosocial support and facilitate public education. These efforts should include awareness campaigns to improve understanding of ACEs, correct misconceptions that frame them as personal failure, and promote early help-seeking behaviors.

## Figures and Tables

**Table 1:** Demographic characteristics of the participants.

No.	Pseudonym	Gender	Born Year	Degree	Occupation	Interview Duration
1	Young	Male	2001	Master	Student	30 min
2	Dong	Male	1999	Master	Student	60 min
3	Xin	Male	1971	Junior high school	Cleaner	30 min
4	Han	Female	2001	Master	Student	30 min
5	Du	Female	1969	Junior high school	Full-time housewife	30 min
6	Chen	Male	1971	Junior high school	Worker	30 min
7	Ming	Male	2001	Bachelor	Student	30 min
8	Dan	Female	1993	Master	Acupuncturist	30 min
9	Chao	Female	2002	Bachelor	Student	30 min
10	Lian	Female	1966	Senior high school	Full-time housewife	30 min
11	Qin	Female	1998	Master	Accounting	30 min
12	Sun	Female	1975	Junior high school	Auxiliary police	30 min
13	Tao	Male	1992	Junior high school	Book designer	30 min
14	Guo	Male	2000	Master	Student	30 min
15	Nu	Female	1975	Bachelor	Physician	30 min
16	Cui	Female	1996	Master	Teacher	30 min
17	Meng	Female	1997	Bachelor	Nurse	30 min
18	Ping	Male	2001	Bachelor	Student	30 min
19	Lei	Male	1990	Doctor	Officer	30 min
20	Yue	Female	1996	Master	Translator	30 min

Note: All participants’ names are pseudonyms.

## Data Availability

The data are not publicly available due to privacy and ethical restrictions.
